# Time to recovery and predictors among admitted preterm neonates in the neonatal intensive care units of public hospitals of Addis Ababa, Ethiopia, 2021

**DOI:** 10.1186/s12887-024-04933-6

**Published:** 2024-07-15

**Authors:** Fekadeselassie Belege Getaneh, Natnael Moges, Dires Birhanu Mihretie, Zebenay Workneh Bitew

**Affiliations:** 1 College of Medicine and Health Sciences, Wollo University, Dessie, Ethiopia; 2 College of Health Sciences, Debre Tabor University, Debre Tabor, Ethiopia; 3 College of Health Sciences, Dilla University, Dilla, Ethiopia; 4 St. Paul’s Hospital Millennium Medical College, Addis Ababa, Ethiopia

**Keywords:** Preterm, Survival, Discharge, Factors, Incidence rate

## Abstract

**Introduction:**

Ethiopia implemented measures to reduce preterm mortality, and much is currently being done to avoid preterm death, yet preterm death remains the top cause of infant death. As a result, evaluating median time of recovery and determinants will provide information to planners and policymakers to design strategies to improve preterm survival.

**Methods:**

Hospital-based retrospective follow-up study was conducted in four selected public hospitals of Addis Ababa from September 2018 to August 2021. Data were collected using a pretested structured questionnaire. Epi-data 4.6 and STATA Version 16 were used for data entry and analysis. Kaplan-Meier survival curve, log-rank test, and median time were computed. To find predictors of time to recovery, a multivariable Cox proportional hazards regression model was fitted, and variables with a p-value less than 0.05 were considered statistically significant.

**Results:**

A total of 466 preterm babies were included in the study of which 261 (56.1%) preterm neonates survived and were discharged from NICUs. The median time to recovery was 10 days (95% CI: 9–12). Low birth weight (Adjusted hazard-ratio [AHR]: 1.91, 95% CI: 1.2–3.06), normal birth weight (AHR: 2.09, 95% CI: 1.16–3.76), late preterm (AHR: 1.91, 95% CI: 1.02–3.55), no hospital-acquired infection (AHR: 2.19, 95% CI: 1.36–3.5), no thrombocytopenia (AHR: 1.96, 95% CI: 1.27–3.02), continuous positive airway pressure (AHR: 0.66, 95% CI: 0.48–0.91), and kangaroo mother care (AHR: 2.04, 95% CI: 1.48–2.81) were found to be independent predictors of time to recovery of preterm babies.

**Discussion/Conclusion:**

The recovery rate was found relatively low. Several predictors of preterm recovery time were discovered in the study. The majority of predictors were preventable or treatable. Therefore, emphasis should be given towards prevention and early anticipation, and management of these predictors. Studies to assess the quality of care and cause of low survival rate of preterm infants are recommended.

**Supplementary Information:**

The online version contains supplementary material available at 10.1186/s12887-024-04933-6.

## Introduction

Preterm neonates are those born before the 37th completed week of pregnancy (259th day), counting from the first day of the last menstrual period [1, 2]. Every year, an estimated 15 million preterm neonates are born worldwide; about 84% are born at 32–36 weeks of gestation, 10% at 28–32 weeks of gestation, and around 5% at 28 weeks of gestation [3].

Preterm complications are the major cause of neonatal death, accounting for 35% of all neonatal death globally (1.1 million deaths per year). The likelihood of death of preterm babies born in Africa is at least 12 times higher than babies born in European Countries. So far, practical, cost-effective care has saved more than three-quarters of preterm babies, and intensive neonatal care could reduce preterm mortality by up to 75% [4, 5].

Even though preterm neonates’ survival has improved dramatically since the introduction of highly specialized intensive care, neonatal deaths in resource-limited settings such as Ethiopia sustained a major public health problem [6]. Prematurity was the leading cause of neonatal deaths in various regions of Ethiopia. In Ethiopia, the neonatal mortality rate was reported to be 30 deaths per 1000 live births in 2019 [7].

Even if the chance of preterm survival depends on birth weight and gestational age, with advanced treatment and care, the likelihood survival of neonates at a gestational age of 23 weeks,25 weeks,28to31 weeks, and 32 to 33 weeks was 17%, 50%, 90%, 95%, respectively, and if delivered above 34 weeks almost as likely as a full-term [8].

To the author’s knowledge, no study has been conducted to assess the recovery time of preterm babies admitted at NICUs and its predictor. Previous studies conducted in Ethiopia and other resource-limited settings sought to establish hospital-based incidence of preterm survival and its association with some maternal characteristics and obstetric complications. [6, 9–11]

Preterm mortality not only tarnishes the country’s image, but it also has terrible social, psychological, and economic consequences for the family and community. As a result, the capacity to accurately anticipate duration of stay in neonatal care is critical for resource planning, service commissioning, and clinician counseling of parents. So, this study aims to determine the median recovery time and predictors among preterm admitted intensive care units of selected public hospitals of Addis Ababa, Ethiopia.

## Methods

### Study design, study area, and study period

An institution-based retrospective follow-up study was conducted among preterm newborns admitted to selected public hospitals in Addis Ababa. The research was conducted from September 15 to October 30, 2021, using records of preterm neonates admitted to NICUs between September 1, 2018, and August 30, 2021. There are twelve government hospitals in the town. Eleven of them have a neonatal unit. The study was conducted at Tikur Anbesa Specialized Hospital, Yekatit 12 Hospital Medical College, St. Peter Specialized Hospital, and Gandhi Memorial Hospital, all were chosen randomly through the lottery method. In these health facilities, continuous life support and comprehensive care are provided for extremely high-risk newborn infants as well as those with complex and critical illnesses.

### Population

The source populations were all preterm neonates who were admitted to the neonatal intensive care units of public hospitals of Addis Ababa and the study population was all preterm neonates admitted in the neonatal intensive care units of selected public hospitals of Addis Ababa from September 2018 to August 30th, 2021.

### Inclusion and exclusion criteria

Between September 2018 and August 30th, 2021, all confirmed preterm deliveries admitted to neonatal intensive care units of public hospitals in Addis Ababa were included in the study. The participants in the study were followed from the time of admission until an event or censorship occurred. This research excluded all preterm infants with incomplete medical records (unknown admission or discharge date) and major congenital abnormalities (congenital anomalies that are not compatible with life).

### Sample size and sampling procedure

**Fig. 1:**
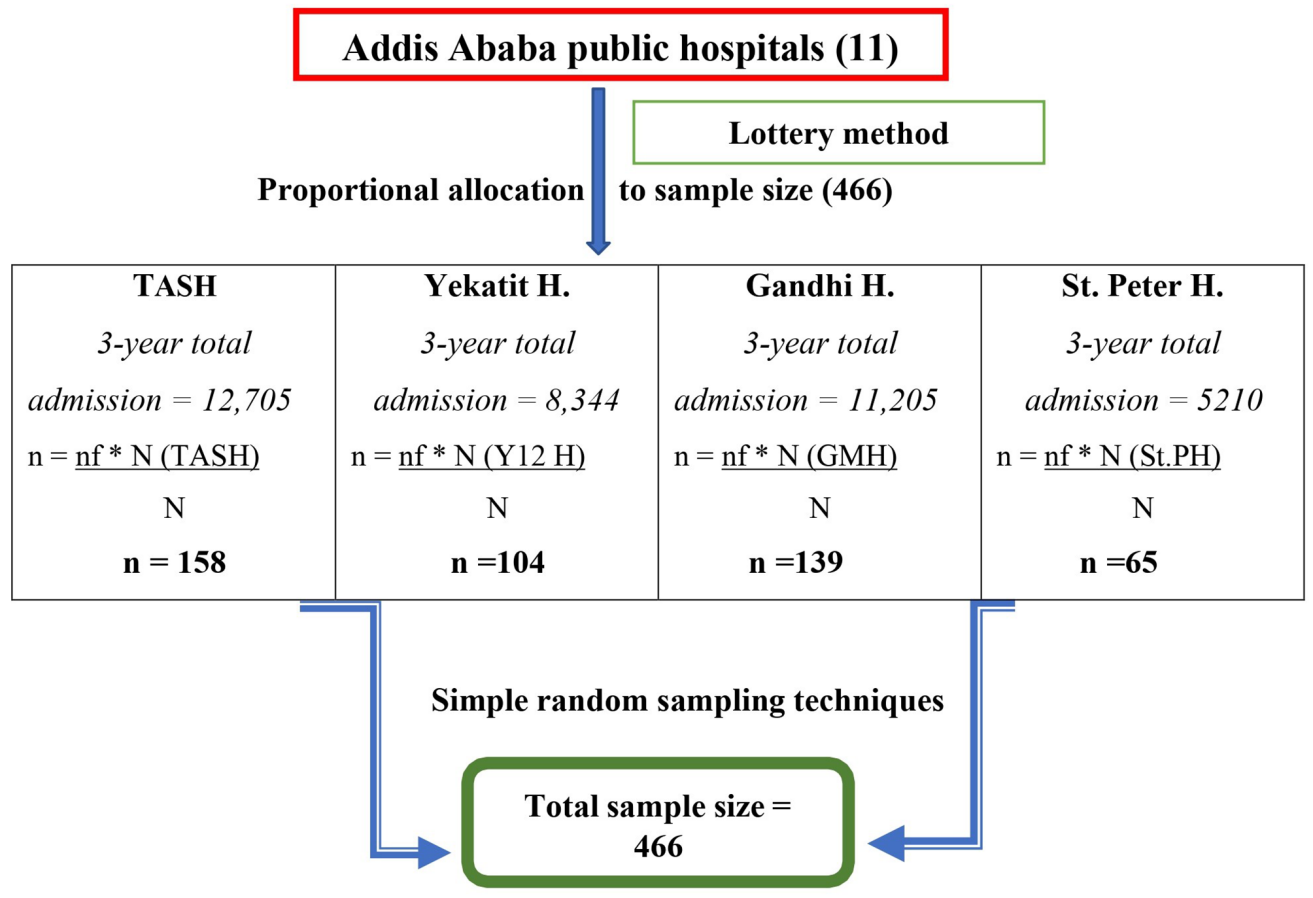
Schematic presentation of sampling procedure on median discharge time and predictors among preterm newborns admitted to NICUs of public hospitals, A.A, Ethiopia, 2021

The sample size was determined using a double population proportion formula by considering birth weight, gestational age, resuscitation, and being married as all these factors predict survival in preterm newborns [11]. Among those predictors, birth weight was found to be an independent predictor that gave a maximum of 438 sample size, by adding 10% for missing data gives a total sample size of 482. The proportional allocation formula was utilized to select study participants from each hospital, considering the total admissions over the course of the three-year study period (Fig. 1). The registration logbook was then used to identify the medical record numbers of preterm newborns. Study participants were then chosen using a computer-generated simple random sampling procedure from the isolated medical record numbers in each facility. Finally, the selected medical charts were reviewed, from September 15 to October 30, 2021.

### Variables

Outcome variable is time to discharge dichotomized as (discharged = 1 and died = 0). Independent variables include, *Socio-demographic and Obstetrics factors* - maternal age, place of residency, gestational hypertension, antepartum hemorrhage, ANC follow up, parity, mode of delivery, place of delivery, oligohydramnios, and chorioamnionitis, *Neonatal related predictors* - Sex, gestational age, birth weight and age at clinical presentation, *Clinical predictors* - Apgar score, hypothermia, asphyxia, hospital-acquired infection, thrombocytopenia, dehydration, *Treatment-related predictors* - CPAP, antibiotics, kangaroo mother care, calcium gluconate and, Aminophylline.

### Operational definitions

*Preterm*: neonates who are diagnosed as preterm either by last normal mensuration period, by Ballard score, or using early ultrasound (20weeks). *Major congenital anomalies* – a wide range of abnormalities of body structure and function that present at birth, like heart defect, neural tube defect, and Down’s syndrome. *Inborn* - refers to babies born at the same facility where they were admitted to the NICU, while *out-born* refers to babies born outside the admitting health facility and subsequently transferred to the NICU. *Kangaroo mother care (KMC)*: in this study, we considered KMC if the baby got continuous type of KMC. *Hospital-acquired infection (HAI)*: an infection acquired in a hospital by a neonate who was admitted for a reason other than admitting health problems or a confirmed common health institution pathogen. *Neonatal sepsis*: is an infection that is usually diagnosed at the time of admission through clinical features and/or laboratory tests such as CBC and C-reactive protein. *Incidence density rate of recovery* – It will be computed by dividing the number of events by total follow-up time in person-days. *Length of stay* – number of days it takes from admission until a baby is discharged from the NICU. *Censored*: Preterm neonates who experienced death (documented by a death summary sheet), were discharged against medical advice (with the caregiver’s consent to leave treatment before recovery), or were referred to other health facilities. *Event* - newborns discharged alive from NICU.

### Data collection tool and procedure

To standardize the data collection, data collectors and supervisors were provided training on the importance of the study, the completion of the checklist, and ethical considerations. Then, according to the eligibility criteria, all research participants’ records were selected, and all available information on patient records was reviewed. Using a standardized data extraction format (Supplemental file I) adapted from the standardized HMIS registration book, and other peer-reviewed articles [9, 11–13], all relevant variables that met the study objectives were collected from patients’ charts. Pretest was carried out on 5% (24 charts) of the study samples to verify data quality. Errors found throughout the verification process were corrected and amended before analysis to ensure that the data abstraction format was by the study objectives. During data management, storage, cleaning, and analysis, all completed data collecting forms were checked for completeness and consistency. The principal investigator also checked for consistency by randomly selecting medical records and cross-checking them for consistency.

### Data processing and analysis

Data were coded, entered, and cleaned in Epi data version 4.6 before being exported to STATA Version 16.0 for further analysis. The data were presented using descriptive and inferential statistics. Log-rank test was performed to assess significant differences in the survival curves across categorical predictors, and Kaplan Meier survival curve was utilized to estimate survival time. For each explanatory variable, a bivariable Cox proportional hazards regression model was fitted. Factors with a p-value less than 0.05 were then fitted to a multivariate Cox regression analysis to find independent predictors of preterm survival. Variables with a P-value less than 0.05 were considered significant predictors of survival of preterm newborns in a multivariable Cox regression analysis. Schoenfeld’s residual test was used to verify the overall Cox-proportional hazard regression assumption. The findings were then summarized and presented in the form of texts, tables, and graphs.

## Result

### **Socio-demographic &** obstetric **characteristics of mothers of study participants**

A total of 482 preterm neonatal records were reviewed, of which 466 (96.7%) were eligible in this study. The majority of the mothers, 407 (87.3%), were between the ages of 20 and 34. The mother’s age ranged from 17 to 40 years old, with a median (IQR) of 28 (6 years). There were 466 (86.05%) mothers from Addis Ababa. More than half of the mothers (56.01%) were multiparous, and 97.21% had antenatal care during their pregnancy. In 246 (52.79%) of the cases, the modes of delivery were spontaneous vaginal delivery, with 207 (44.4%) being cesarean delivery (C-section) and assisted vaginal delivery accounted for the remaining 2.8% of deliveries. Nearly half of the mothers, 229 (49.14%), gave birth at the same facility where their infant was admitted, while 11 (2.4%) of mothers gave birth at home. 127 (27.25%) of pregnant women with Obstetrics & Medical Complications had Hypertension during their pregnancy, and 37 (7.94%) of moms had an antepartum hemorrhage. Nineteen mothers (4.1%) developed oligohydramnios, and 13 (2.79%) showed signs of chorioamnionitis (Table 1).

**Table 1:** Socio-demographic and obstetrics characteristics of a pregnant mother who had admitted preterm neonates at NICUs of Addis Ababa public hospitals, (*n* = 466)

Covariates	Category	Total Number (%)	Status	
			**Survived** Number (%)	Censored Number (%)
Maternal Age	< 20 years	17(3.64)	8(3.06)	9(4.39)
	20–34 years	407(87.3)	226(86.59)	181(88.29)
	> 34 years	42(9.01)	27(10.34)	15(7.31)
Place of residency	Addis Ababa	401(86.1)	223(85.4)	178(86.8)
	Out of Addis Ababa	65(13.9)	38(14.6)	27(13.2)
Parity	Primi-parous	205(44)	108(41.4)	97(47.3)
	Multiparous	261(56)	153(58.6)	108(52.7)
ANC follow up	No	13(2.8)	3(1.1)	10(4.9)
	Yes	453(97.2)	258(98.9)	195(95.1)
Place of delivery	Out born	226(48.5)	134(51.3)	92(44.9)
	Inborn	229(49.1)	123(47.1)	106(51.7)
	home	11(2.4)	4(1.5)	7(3.4)
Mode of delivery	SVD *	246(52.8)	136(52.1)	110(53.7)
	C/S delivery	207(44.4)	116(44.4)	91(44.4)
	Assisted delivery	13(2.8)	9(3.4)	4 (2)
Types of pregnancy	singleton	343(73.6)	189(72.4)	154(75.1)
	multiple	123(26.4)	72(27.6)	51(24.9)
Obstetrics & medical conditions	PIH*	127(27.3)	47 (18)	80(39)
	APH**	37(7.9)	18(6.9)	19(9.3)
	Oligohydramnios	19(4.1)	10(3.8)	9(4.4)
	chorioamnionitis	13(2.8)	7(2.7)	6(2.9)

PIH*- pregnancy induced hypertension APH**-Antepartum hemorrhage

### Neonatal characteristics of the study participants

Out of the 466 cohorts, (53.86%) were males. When comparing the mean duration of neonates’ presentations on NICUs, it was found that out-born preterm babies (23.8 ± 5.8 h) and died babies (14.6 ± 4.7 h) had a longer duration compared to inborn babies (2.5 ± 0.8 h) and preterm neonates who survived (11.6 ± 3.6 h). In the group of surviving babies, 56.3% and 82.3% of preterm infants had APGAR scores (> 7) within the normal range at the first and fifth minutes of life, respectively. However, among babies who unfortunately did not survive, 41.9% and 72.2% had normal APGAR scores at the first and fifth minutes of life, respectively. Nearly half (47.31%) of the babies were born before 34 weeks of gestational age and the majority (88%) of the baby were appropriate for their gestational age. Four hundred twenty (90%) of babies had low birth weight (< 2500 gram) at the time of birth.

### Clinical characteristics of the study participants

The most common additional medical diagnosis among preterm infants at admission was early-onset sepsis (78.9%), respiratory distress (71%), hypothermia (64.8%), and prenatal asphyxia (28.8%). A total of 331 (71%) newborns had a second medical diagnosis in addition to their admission diagnosis. Hyperbilirubinemia, thrombocytopenia, apnea of prematurity, and suspected necrotizing enterocolitis 49.14%, 31.97%, 28.33%, and 20.6%, were additional medical diagnoses after admission during their hospital stays, respectively. 109 newborns (23.4%) showed evidence of dehydration, and 24.25% showed signs of hospital-acquired infection (HAI).

### Treatment characteristics of the study participants

The majority (94.2%) of babies took antibiotics and 295 (63.3%) of preterm babies were put on CPAP as management within their hospital stay. The chance of surviving in babies who got kangaroo mother care was almost double (69% vs. 35%) compared to those who were not put on KMC. More than half (61.59%) babies got calcium gluconate as prophylaxis and management and 120 (25.75%) apneic babies were treated with aminophylline during their hospital stay.

### Survival status of preterm neonates

**Fig. 2:**
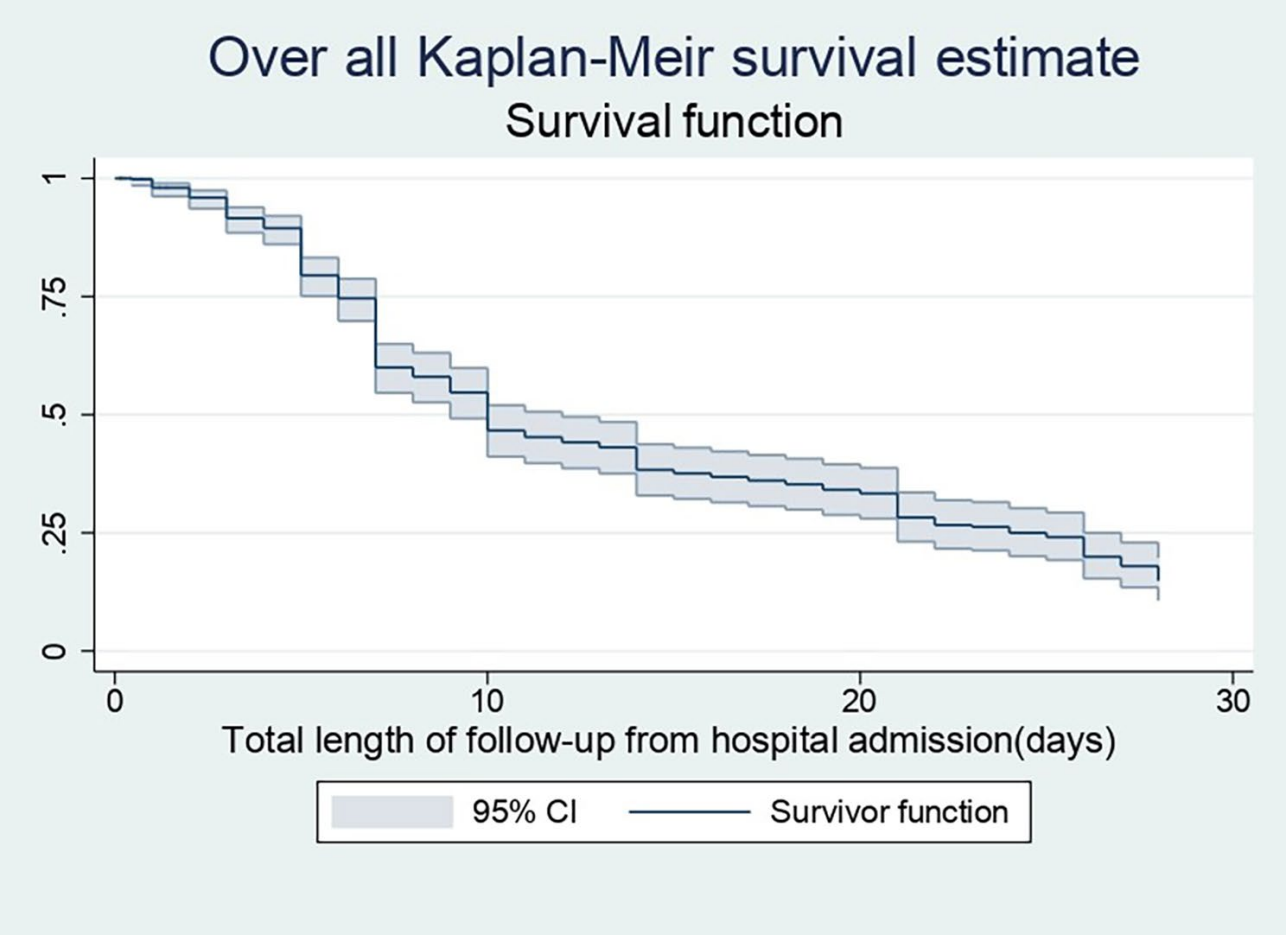
Overall Kaplan-Meier survival estimate of preterm neonates admitted in Addis Ababa public hospitals from 2018–2021, Addis Ababa, Ethiopia, 2021

Neonates were followed for a total of 4506 neonate days, ranging from 3 h to 28 days, with a median discharge time of 10 days (95% CI: 9–12). Throughout the cohort, 261 (56.1%) preterm neonates were survived and discharged, while 205 (43.9%) died. Furthermore, the majority of the deaths among the neonates (60%) were documented within the first five days of birth. The overall incidence density rate of recovery was 6 per 100 neonatal-days of observation (95% CI: 0.05–0.07), and the estimated cumulative probability of discharge at 1,3,7,14,21, and 28 days was 97%, 85%, 52%, 25%, 18% and 7%, respectively (Fig. 2).

### Time to recovery among different groups of preterm neonates

**Fig. 3:**
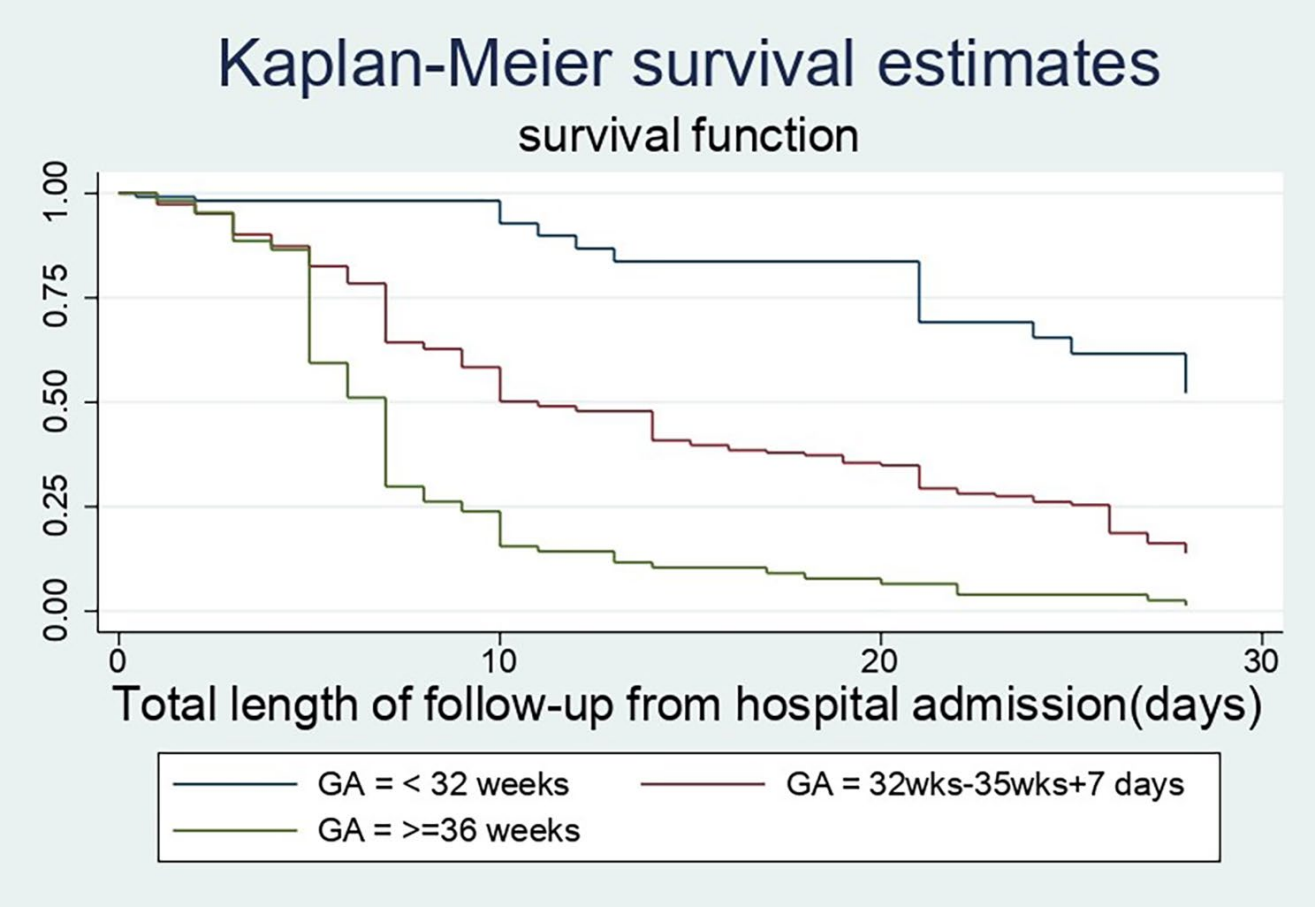
The KM survival curves compare discharge time with different gestational ages among preterm babies who were admitted at NICUs of Addis Ababa public hospitals, Ethiopia, from 2018-to 2021

**Fig. 4:**
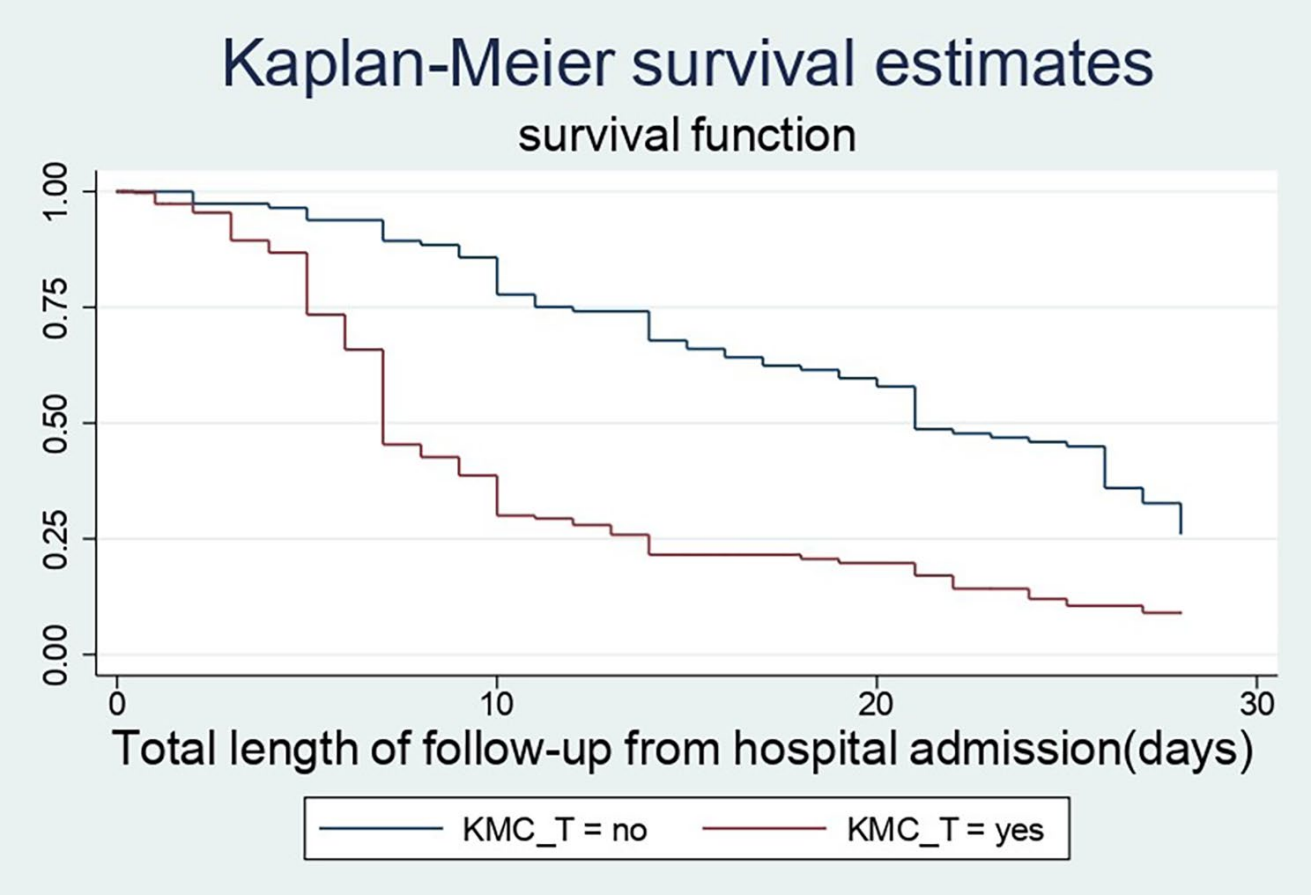
The KM survival curves compare discharge time of kangaroo mother care among preterm babies who were admitted at NICUs of Addis Ababa public hospitals, Ethiopia, from 2018–2021

**Fig. 5:**
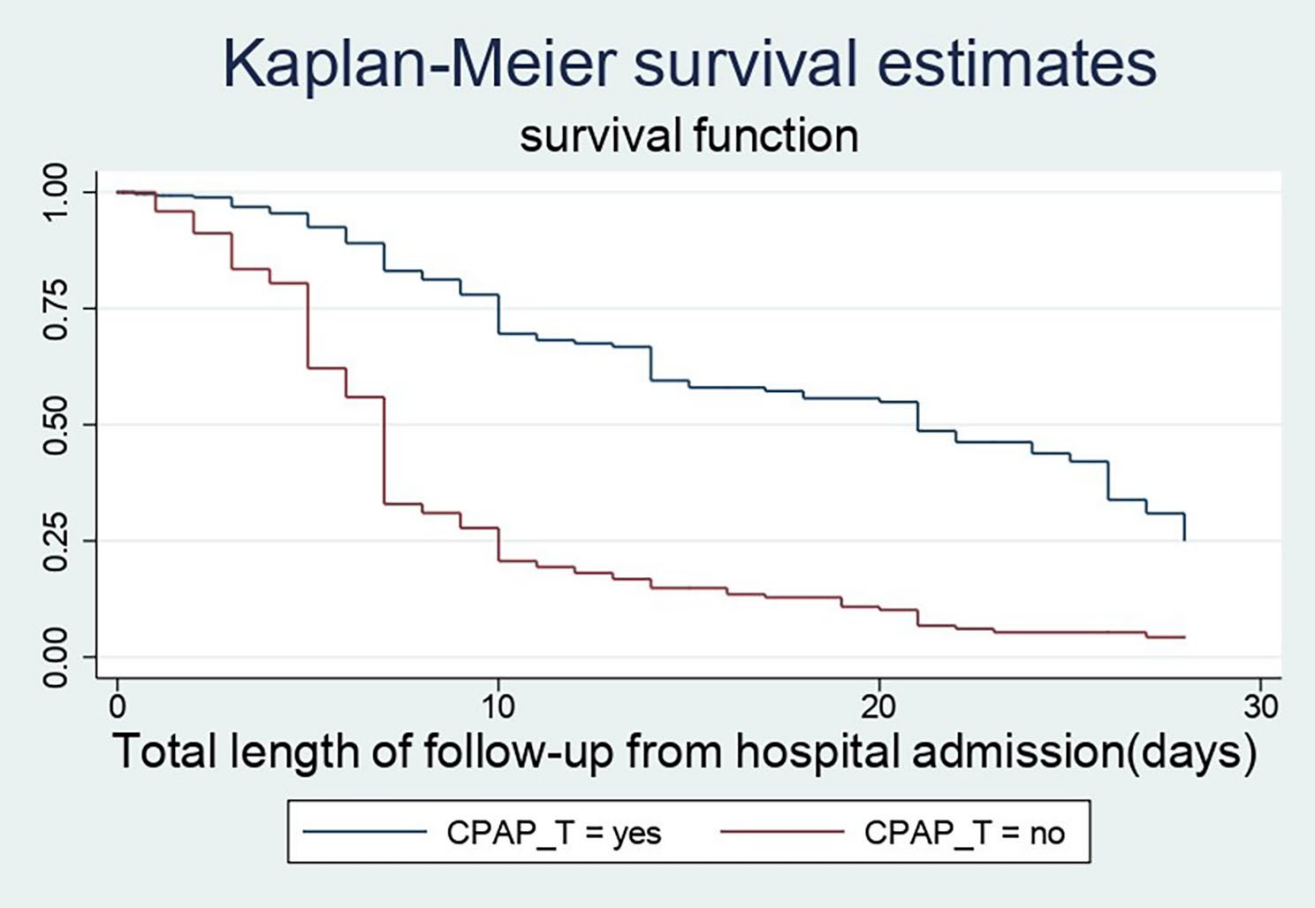
The KM survival curves compare discharge time of CPAP therapy among preterm babies who were admitted at NICUs of Addis Ababa public hospitals, Ethiopia, from 2018–2021

In this study, preterm babies with very low birth weight had a substantially longer recovery period than those with low or normal birth weight (28 days Vs 10 and 7 days), respectively. Preterm newborns that did not develop thrombocytopenia throughout their hospital stay had a better likelihood of recovering faster than their counterparts (7 days 95% CI: 7–9) and (28 days 95% CI: 25–28). Furthermore, the median recovery time for preterm neonates who had a hospital-acquired infection was approximately four times longer than for those who did not have a sign of infection throughout their hospital stays (28 days Vs 7 days). When compared to their counterparts, late preterm infants, an infant treated with continuous positive airway pressure and kangaroo mother care had a higher chance of being discharged earlier. (Figures 3, 4 and 5)

### Predictors of survival status of preterm neonates

In bi-variable Cox-regression, birth weight, gestational age, hypothermia at admission, respiratory distress syndrome, sepsis, perinatal asphyxia, necrotizing enterocolitis, hospital-acquired infection, neonatal hyperbilirubinemia, apnea of prematurity, thrombocytopenia, dehydration, CPAP, and kangaroo mother care were found to be significant at p-value < 0.05 and fitted for multivariable Cox-regression analysis. Low birth weight, normal birth weight, late preterm, hospital-acquired infection, thrombocytopenia, CPAP, and kangaroo mother care were identified predictors of preterm discharge time in multi-variable analysis.

Low birth weight and normal birth weight preterm newborns had 1.9 times and 2 times better chances of being discharged earlier from the NICU than very low birth weight preterm babies (AHR: 1.91, 95% CI: 1.2–3.06) and (AHR: 2.09, 95% CI: 1.16–3.76), respectively. Late preterm newborns, on the other hand, had a 1.9 times better chance of being discharged than early preterm babies (AHR: 1.91, 95% CI: 1.02–3.55).

The study findings indicate that preterm babies who did not develop thrombocytopenia and hospital-acquired infection during their hospital stay were discharged earlier compared to their counterparts. Specifically, the discharge rate was 1.9 times higher (AHR: 1.96, 95% CI: 1.27–3.02) for babies without thrombocytopenia and 2.2 times higher (AHR: 2.19, 95% CI: 1.36–3.5) for babies without hospital-acquired infection.

When compared to those who did not require CPAP, neonates who required CPAP had longer discharge times (AHR: 0.66, 95% CI: 0.48–0.91), and neonates who got kangaroo mother care had two times higher chance of discharge earlier than their counterparts (AHR: 2.04, 95% CI: 1.48–2.81). **(**Table 2**).** Schoenfeld’s residual test (global test) was used to verify the proportional hazard assumption. The results showed that all of the model’s individual variables met PH assumptions (p-value > 0.05) and (Global test for Cox proportional hazard P-Value = 0.393 > 0.05).

**Table 2:** Bivariate and Multivariable Cox regression analysis results of preterm babies who were admitted at NICUs of Addis Ababa public hospitals, Ethiopia, 2021 [*n* = 466]

Covariates	CHR (95% CI)	*P*-value	AHR (95% CI)	*P*- value
**Birth weight**				
< 1500 gram	1		1	
1500 gm– 2499 gm	3.64(2.36–5.61)	0.000	**1.91(1.2–3.06)**	0.006*
*≥* 2500 gram	6.95(4.15–11.65)	0.000	**2.09(1.16–3.76)**	0.014*
**Gestational Age**				
< 32 weeks	1		1	
32 weeks – 35 + 7 weeks	3.74(2.2–6.36)	0.000	1.36(0.76–2.43)	0.289
36 weeks – 37 weeks	8.65(4.98–15.05)	0.000	**1.91(1.02–3.55)**	0.040*
**Hypothermia at admission**				
Yes	1		1	
No	1.81(1.42–2.31)	0.000	0.93(0.85–1.71)	0.292
**Respiratory distress syndrome**				
Yes	1		1	
No	1.91(1.49–2.44)	0.000	1.24(0.7–1.24)	0.659
**Sepsis**				
Yes	1		1	
No	1.58(1.2–2.07)	0.001	1.46(0.68–1.96)	0.112
**Perinatal asphyxia**				
Yes	1		1	
No	1.38(1.01–1.89)	0.040	1.11(0.78–1.59)	0.533
**Necrotizing enterocolitis**				
Yes	1		1	
No	7.31(3.88–13.78)	0.000	1.99(0.99–3.98)	0.051
**Hospital acquired infection**				
Yes	1		1	
No	5.51(3.67–8.24)	0.000	**2.19(1.36–3.50)**	0.001 *
Table 2: Bivariate and Multivariable Cox regression analysis results of preterm babies who were admitted at NICUs of Addis Ababa public hospitals, Ethiopia, 2021 [*n* = 466]. **Continue ….**				
**Covariates**	**CHR (95% CI)**	**P-value**	**AHR (95% CI)**	**P- value**
**Neonatal hyperbilirubinemia**				
Yes	1		1	
No	2.05(1.6–2.63)	0.000	1.07(0.81–1.41)	0.634
**Apnea of prematurity**				
Yes	1		1	
No	4.22(2.54–7.01)	0.000	0.94(0.53–1.67)	0.848
**Thrombocytopenia**				
Yes	1		1	
No	4.62(3.2–6.68)	0.000	**1.96(1.27–3.02)**	0.002*
**Dehydration**				
Yes	1		1	
No	4.58(2.67–7.86)	0.000	1.62(0.94–2.78)	0.079
**Continuous positive airway pressure**				
No	**1**		**1**	
Yes	0.28(0.22–0.37)	0.000	**0.66(0.48–0.91)**	0.011*
**kangaroo mother care**				
No	1		1	
Yes	2.78(2.09–3.69)	0.000	**2.04(1.48–2.81)**	0.000*

***NB: -*** *Significant (P-value < 0.05), **significant (p-value < 0.01) and HR = 1 is reference variable

## Discussion

Estimating the median survival time and identifying predictors of survival for preterm newborns is critical for preterm neonatal management and care, especially in resource-limited settings. From the total 466 preterm babies enrolled in this study, 56.1% of neonates were survived and discharged from the NICUs. The findings of this study are lower than those of studies conducted in Ethiopia [6] and Kenya [14] which found that 70.69% and 60.6% of preterm neonates were discharged, respectively. Beyond, the differences in the scope of the study, study sites, and study design. The majority of our study areas were teaching and specialized hospitals. Due to this nearly all admitted preterm babies were severely ill or had additional medical diagnoses additional to prematurity because of born from mothers who had medical and obstetric complications during their pregnancy and labor time. The overall survival rate of this study was greater than a retrospective cross-sectional study conducted in Felege-hiwot specialized hospital (49.1%), which was a one-year study conducted in a single health facility [13].

In our study, the median time of survival or hospitalization was 10 days. This is earlier than Dire-Dawa Public Hospitals (14.3 days) [15] and Mizan Tepi University Teaching Hospital (15 days) [16]. However, it was 3days longer than the Gondar comprehensive specialized hospital (7 days) [12]. This discrepancy may be due to the availability of highly specialized professionals and a difference in the scope of care provided by institutions for critically ill preterm infants.

Several studies have found that preterm newborns with normal birth weight and late preterm babies had a better prognosis [14, 16, 17]. This study also revealed normal birth weight and late preterm babies have a better probability of surviving and being discharged from the NICU. This could be explained by the fact that newborns with normal birth weight and those born late had a better cardiopulmonary transition, surfactant production, and brown fat than their counterparts [18]. This may improve survival by reducing the risk of complications such as respiratory problems and hypothermia.

The hazard of survival for preterm neonates who had normal platelet count during their stay was 1.96 times higher compared to neonates who had thrombocytopenia. This may be related to the fact that thrombocytopenia was found in the majority of preterm babies, babies who needed to be in NICUs due to sickness, and babies born from mothers who had prenatal complications [19, 20]. Even if a platelet transfusion is the mainstay of management for thrombocytopenic preterm, there is a gap in accessibility, appropriate storage, and proper administration. This could prolong the recovery and discharge time.

The current study showed that preterm babies who showed no signs of hospital-acquired infection (HAI) during their hospital stay had a two-fold better chance of being discharged. HAI are frequent complications in newborns with medical disorders requiring prolonged hospitalization. Premature neonates have a high risk of infection because of their lack of protective maternal antibodies, underdeveloped innate immunity, and fragile, easily damaged skin [21, 22]. Furthermore, the poor intervention of infection control protocol, poor diagnosis, and management of HAI in health facilities may contribute to prolong hospitalization.

Unexpectedly, preterm neonates who received CPAP as a treatment were less likely to be discharged earlier than those who did not. This finding contradicts the findings of a Tanzanian randomized trial study [23]. CPAP can improve overall outcomes and shortened NICU stays in smaller newborns [24]. However, in real-life clinical situations, CPAP machines are scarce, and complications such as nasal bleeding, gastric distension, and oral feeding delays are poorly managed. This could cause the discharge to be delayed.

Preterm neonates who received Kangaroo Mother Care (KMC) demonstrated a faster recovery and were discharged earlier compared to their counterparts. This study was in line with the study done at the University of Gondar Hospital [12], Mizan-Tepi Hospital [16], and Axum [25]. This could be due to a lower the risk of hypothermia, apnea, and delayed breastfeeding initiation [26, 27]. This reduced risk contributed to positive outcomes, including a lower incidence of preterm complications such as hypoglycemia, severe hypothermia, and cardiopulmonary instability. Consequently, infants who received KMC had shorter hospital stays and a decreased likelihood of mortality.

The limitations of the study are this study’s focus on teaching and specialized hospitals, where admitted preterm babies were predominantly severely ill or had additional medical diagnoses, may limit the generalizability of the findings and impact survival time. Since it is a retrospective study, the study did not address the probable service-related predictors and other maternal predictors. Finally, excluding the incomplete medical records may also contribute to selection bias.

## Conclusion

In this study, the overall survival of preterm neonates was relatively low, and most of the study participants had a long duration of hospital stay. Low birth weight, normal birth weight, late preterm, hospital-acquired infection, thrombocytopenia, CPAP, and kangaroo mother care were identified predictors of preterm recovery time. The majority of these predictors are preventable or treatable. As a result, prevention and early detection, as well as management of these factors, should be prioritized. A study to assess the quality of care and cause of the very low survival rate of preterm infants is recommended.

### Electronic supplementary material

Below is the link to the electronic supplementary material.

Supplementary Material 1

## Data Availability

The data sets used and/or analyzed during the current study are available from the corresponding author on reasonable request.

## Abbreviations

AAU: Addis Ababa University
AHR: adjusted hazard ratio
ANC: antenatal care
APGAR: appearance, pulse, grimace, activity, respiration
CPAP: Continuous Positive Airway Pressure
GA: gestational age
HMIS: health management information system
KMC: kangaroo mother care
NEC: necrotizing enterocolitis
NICU: Neonatal Intensive Care Unit
