# Supplementary material for: Time to recovery and predictors among admitted preterm neonates in the neonatal intensive care units of public hospitals of Addis Ababa, Ethiopia, 2021
Source: BMC Pediatr. 2024 Jul 15;24:452. doi: 10.1186/s12887-024-04933-6 (PMC11247717; doi:10.1186/s12887-024-04933-6)
Supplement: Supplementary file 1 — Supplementary Material 1 [file 12887_2024_4933_MOESM1_ESM.pdf]

Supplemental file I: Data Extraction Tool

Part I: Socio-demographic and Obstetrics characteristics of a pregnant mothers of admitted preterm neonates

No	Maternal socio demographic Characteristics	Response	Skip to
101	Age of the mothers	___________Year	
102	Marital status	1. Single<br>2. Married<br>3. Divorced<br>4. Widowed	
103	Place of Residency	1. Urban<br>2. Rural	
104	Parity	1. Primi-parous<br>2. Multiparous	
105	ANC follow Up	1. Yes<br>2. No	107
106	If yes, how many times?	_______________	
107	Type of pregnancy	1. Singleton<br>2. Multiple	
108	If the pregnancy is multiple	1. First baby<br>2. Second baby above	
109	Mode of Delivery/delivery method/	1. SVD<br>2. Instrumental delivery<br>3. C/S	
110	Place of delivery	1. Outborn<br>2. Inborn	
111	Risk of preterm birth	1. Yes<br>2. No	113
112	If yes, risk of preterm birth (multiple response)	1. PPROM<br>2. APH<br>3. Preeclampsia/Eclampsia<br>4. Oligo/polyhydramnios<br>5. Other specify…..	
113	Does the mother diagnose with chronic disease	1. Yes<br>2. No	201
114	If yes which one of the medical problems (multiple response)	1. Diabetes<br>2. Hypertension<br>3. Anemia<br>4. Tuberculosis<br>5. HIV/AIDS<br>6. Others specify ……..	

Part II: Preterm Sociodemographic, medical problem and treatment related characteristics

	**Preterm demographic characteristics**	**Response**	**Skip to**
201	Sex	1. Male<br>2. Female	
202	Age at admission	___________hr./day	
203	Gestational age	____________Week	
204	Birth Weight	____________gram.	
205	Weight for gestational age	1. AGA<br>2. LGA<br>3. SGA	
301	APGAR score at birth	1. 1st minute _____<br>2. 5th minute _____	
302	Diagnosis at admission (multiple response)	1. Hypothermia<br>2. RDS<br>3. EONS<br>4. PNA<br>5. NHB(Jaundice)<br>6. Meningitis<br>7. Other specify ……..	
303	Does the neonate develop new medical problem between the follow up	1. Yes<br>2. No	401
304	New medical problems between the follow up (multiple response)	1. HAI<br>2. NEC<br>3. NHB (Jaundice)<br>4. Apnea of prematurity<br>5. Hypothermia<br>6. Hypoglycemia<br>7. Thrombocytopenia<br>8. DHN<br>9. Other specifies….	
401	Does the neonate eligible to CPAP	1. Yes<br>2. No	403
402	Time of initiation of CPAP	1. Since delivery<br>2. At admission<br>3. After admission	
403	Type of CPAP the neonates put on	1. Dimedica<br>2. Home Grown	
404	Does the neonate receive antibiotics	1. Yes<br>2. No	406

405	If yes time of antibiotics initiation in reference to admission	______________hr./day	
406	Does the neonate start feeding	1. Yes<br>2. No	408
407	If yes, feeding initiation at the age of	_______hr./day	
408	Does the neonate receive KMC	1. Yes<br>2. No	
501	Length of stay in the hospital	_________hr./day	
502	Outcome of the neonate	1. Recovered<br>2. Censored	
503	If the answer to question number 417 is censored, reason of being censored	1. Died<br>2. Left against medical advice<br>3. Transferred/referred<br>4. >28days age at follow up	417
504	If died immediate cause of death (multiple response)	___________________	
Thanks a Lot!			
